# Comparison of radiological characteristics between diffuse idiopathic skeletal hyperostosis and ankylosing spondylitis: a multicenter study

**DOI:** 10.1038/s41598-023-28946-w

**Published:** 2023-02-01

**Authors:** Takuya Takahashi, Toshitaka Yoshii, Kanji Mori, Shigeto Kobayashi, Hisashi Inoue, Kurisu Tada, Naoto Tamura, Takashi Hirai, Nobuhiro Sugimura, Narihito Nagoshi, Satoshi Maki, Keiichi Katsumi, Masao Koda, Kazuma Murata, Kazuhiro Takeuchi, Hiroaki Nakashima, Shiro Imagama, Yoshiharu Kawaguchi, Masashi Yamazaki, Atsushi Okawa

**Affiliations:** 1 Department of Orthopedic Surgery, Tokyo Medical and Dental University, 1-5-45 Yushima, Bunkyo-ku, Tokyo 113-8510 Japan; 2 Department of Orthopedic Surgery, Shiga University of Medical Science, Ōtsu, Shiga Japan; 3 Department of Internal Medicine, Juntendo University Koshigaya Hospital, Juntendo University School of Medicine, Koshigaya, Saitama Japan; 4 Department of Orthopedic Surgery, Juntendo University School of Medicine, Bunkyo-ku, Tokyo Japan; 5 Department of Internal Medicine and Rheumatology, Juntendo University School of Medicine, Bunkyo-ku, Tokyo Japan; 6 Tokyo Medical and Dental University School of Medicine, Bunkyo-ku, Tokyo Japan; 7 Department of Orthopedic Surgery, School of Medicine, Keio University, Shinjuku-ku, Tokyo Japan; 8 Department of Orthopedic Surgery, Graduate School of Medicine, Chiba University, Chiba, Chiba Japan; 9 Department of Orthopedic Surgery, Niigata Central Hospital, Niigata, Niigata Japan; 10 Department of Orthopedic Surgery, Faculty of Medicine, University of Tsukuba, Tsukuba, Ibaraki Japan; 11 Department of Orthopedic Surgery, Tokyo Medical University, Shinjuku-ku, Tokyo Japan; 12 Department of Orthopedic Surgery, National Hospital Organization Okayama Medical Center, Okayama, Okayama Japan; 13 Department of Orthopedic Surgery, Nagoya University Graduate School of Medicine, Nagoya, Aichi Japan; 14 Department of Orthopedic Surgery, Faculty of Medicine, University of Toyama, Toyama, Toyama Japan

**Keywords:** Skeleton, Ankylosing spondylitis, Skeleton

## Abstract

To evaluate the radiological differences between diffuse idiopathic skeletal hyperostosis (DISH) and ankylosing spondylitis (AS) using whole spine computed tomography (CT), including the spine and sacroiliac joint (SIJ). The ossification and bridging of spinal ligament and fusion of the facet joint and SIJ were evaluated in 111 patients who were diagnosed with DISH and 27 patients with AS on the whole spine CT. The number of anterior bridging and shape of bridging (candle-wax-type/ smooth-type) were also evaluated. We further evaluated patients with DISH and AS by matching their age and sex. Complete SIJ fusion was more common in AS, whereas anterior and posterior bony bridging around SIJ was more common in DISH. However, 63% of patients with DISH had a partial or complete fusion. In spinal anterior bony bridging, the majority of patients with AS had the smooth-type, whereas those with DISH had the candle-wax-type. However, some of the patients with DISH (11%) had smooth-type. Intervertebral facet joint fusion is more common in AS. The number of anterior spinal bony bridging was greater in AS than in DISH, especially in the lumbar spine. These results are useful in differentiating DISH from AS and should therefore be considered when making a diagnosis.

## Introduction

Diffuse idiopathic skeletal hyperostosis (DISH) is characterised by calcification and ossification of the anterior vertebral body and peripheral entheses, leading to the bony bridging of multiple vertebral bodies^1^. The prevalence varies between 2.9% in the Asian population aged > 50 years to 42.0% in European men aged > 65 years^2–4^. The reported risk factors for DISH are old age, male gender and metabolic factors such as obesity, hypertension and type 2 diabetes mellitus^5,6^.

To diagnose DISH, various criteria have been used in the clinical setting. Of these, Resnick criteria are the most commonly used^7^, which diagnoses DISH in the spine based on radiographic features based on (1) ‘flowing’ ossification of at least four contiguous vertebral bodies, 2) relative preservation of the intervertebral disc space and 3) absence of apophyseal joint ankylosis and sacroiliac joint (SIJ) erosion, sclerosis, or intraarticular osseous fusion^8^. DISH is also characterised by swelling ossification of the anterior longitudinal ligament^1^. As the number of bone bridging of the vertebrae increases, spinal ankylosis becomes severe, which sometimes causes unstable three-column spinal fracture by minor trauma^9^.

Ankylosing spondylitis (AS) is also known to cause severe spinal ankylosis and is characterised by a chronic inflammatory disease and the presence of the HLA-B27 antigen^1,10,11^. The prevalence varies 0.03–1.8%^12,13^. Symptoms usually appear in the 20–30’s and rarely occur after the age of 40 years^1^. The modified New York criteria show that AS diagnosis is based on radiographic features, including sacroiliitis grade ≥ 2 bilaterally or grade 3–4 unilaterally^14^. AS is also characterised by annulus fibrosus ossification and adjacent vertebral body bridging anteriorly and laterally known as a ‘bamboo spine’^1^.

Although the pathological characteristics of AS are different from that of DISH, patients with DISH may radiologically present with SIJ fusion and/or smooth bony bridging of the vertebrae in clinical practice^1,15,16^. However, no previous reports have compared radiological features of DISH and AS using spinopelvic computed tomography (CT), including the whole spine and SIJ. Moreover, no studies have specifically focused on the characteristics and degrees of SI fusion and spinal ankylosis. Hence, this study aimed to identify the difference in the reconstructed whole spine CT images between DISH and AS to radiologically differentiate the disease from each another.

## Materials and methods

The Ethics Committee of Tokyo Medical and Dental University Hospital approved this study (M2020-235) and informed consent was waived since it was a retrospective, anonymized study. All methods were carried out in accordance with relevant guidelines and regulations.

### Study design and population

This study was conducted by the Japanese Multicenter Research Organisation for Ossification of the Spinal Ligament with the assistance of the Japanese Ministry of Health, Labour and Welfare. This study was performed using ossification of the posterior longitudinal ligament whole spine CT database, in which 50% of the patients have co-existing DISH^17,18^. DISH was diagnosed with anterior longitudinal ligament ossification in at least four contiguous vertebral bodies as previously reported^17,18^. Previous reports^15,19,20^ demonstrated that pelvic findings were excluded from the diagnostic criteria. Patients with AS were retrospectively enrolled in three institutions. Rheumatologists diagnosed AS using the modified New York criteria^14,20^. The inclusion criteria were as follows: age > 20 years; and CT images available to determine the location of ossification, bony bridging, and union in the spine and SIJ. The CT images were obtained with 0.5–1.0 mm thick sections using a 64 or 80-row multi-detector unit. The acquisition parameters were 120–150 kV, using auto exposure control.

This study included 138 patients: 111 with DISH (83 males and 28 females; 68.0 ± 11.8 years) and 27 with AS (21 males and 6 females; 47.0 ± 12.5 years). In patients with AS, the duration of symptoms was an average of 18.7 ± 10.0 years. In clinical data, 82% of patients with AS were HLA-B27 positive and 36% were C-reactive protein positive (≥ 0.3 mg/dL). Regarding the medical treatment, 75% of patients with AS used biologics (46%: adalimumab, 26%: infliximab, and 3%: golimumab). We further evaluated patients with DISH and AS by matching their age and sex (DISH: 13 males, 4 females; AS: 13 males, 4 females).

### Measured data

**Figure 1:**
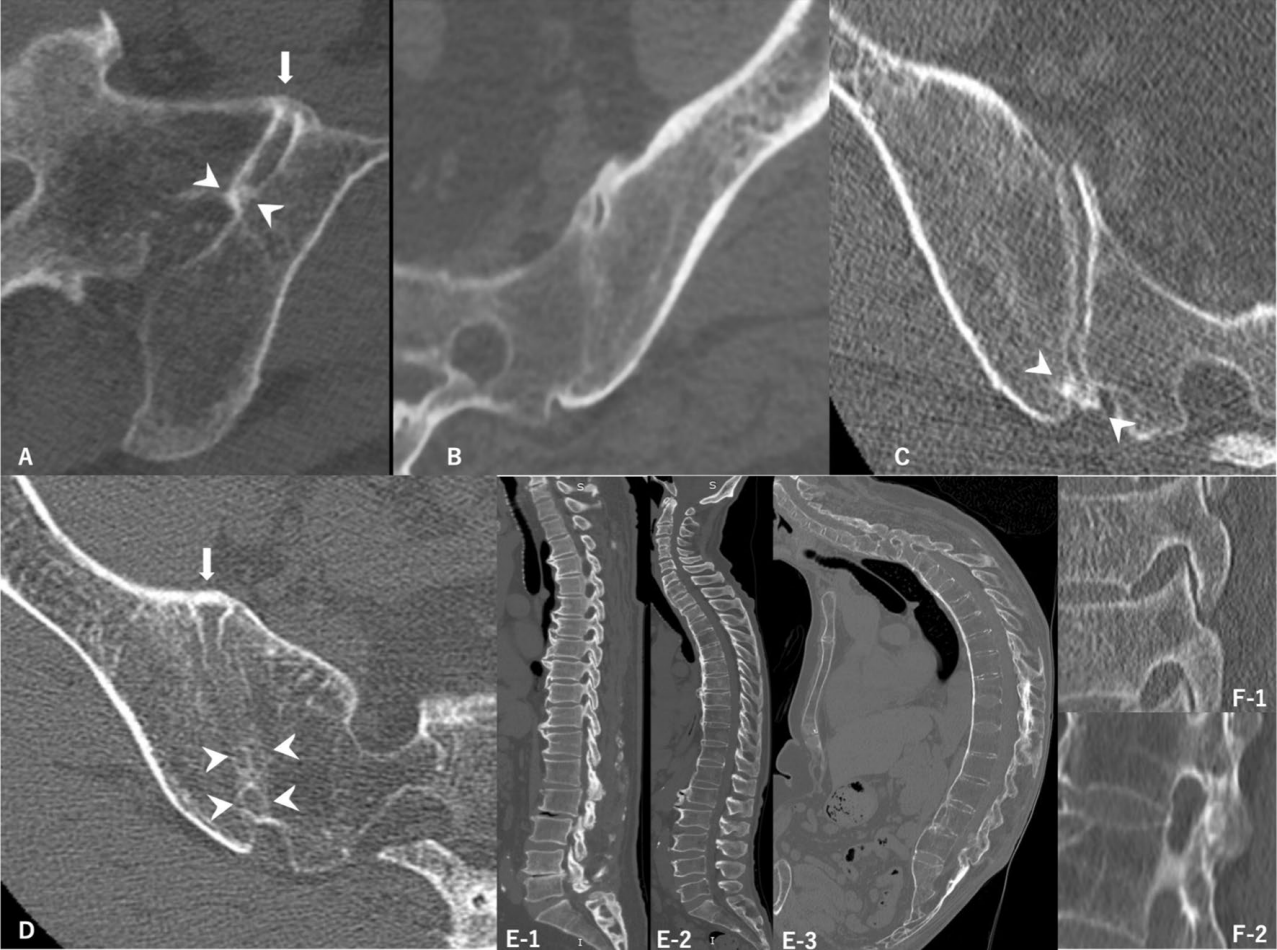
CT images of the spine and SIJ. (**A**) Axial CT image of SIJ shows anterior bony bridging (arrow) and partial fusion of joint (arrowhead). (**B**) Axial CT image of SIJ shows complete joint fusion. (**C**) Axial CT image of SIJ shows posterior bony bridging (arrowhead). (**D**) Axial CT image of SIJ shows anterior bony bridging (arrow) and entheseal bony bridging (arrowhead). (**E**-1) Sagittal CT image of the whole spine shows 70–100% candle-wax-type. (**E**-2) Sagittal CT image of the whole spine shows 30–70% candle-wax-type. (**E**-3) Sagittal CT image of the whole spine shows 0–30% candle-wax-type. (**F**-1) Sagittal CT image of the facet shows no fusion. (**F**-2) Sagittal CT image of the facet shows fusion.

Demographic data including age and sex were collected. Furthermore, radiological data of SIJ were collected, such as joint fusion, anterior bony bridging, posterior bony bridging and entheseal bony bridging, which were defined as previously described^19^ (Fig. 1A–D). Joint fusion was defined as a transverse bony projection within the SIJ that connects the sacrum and ilium^19^. SIJ fusion was classified into none, one-side partial, both sides partial, one-side complete and another side partial and both sides complete. Complete SIJ fusion was defined as intraarticular bony bridging observed at approximately the whole SIJ area on axial CT images. Partial fusion was defined as intraarticular bony bridging observed at the part of the SIJ area. Anterior bony bridging was defined as an arched bony projection beyond the anterior margin of the SIJ, bridging the ilium and sacrum and without involving the intraarticular part of the joint^19^. Posterior bony bridging was defined as an arched bony projection beyond the posterior margin of the SIJ, bridging the ilium and sacrum and without involving the intraarticular part of the joint^19^. Entheseal bony bridging was defined as a transverse bony projection within the posterior sacroiliac ligaments that connect the sacrum and ilium^19^. Bridging is classified into none, one-side and both sides. Two examiners evaluated the CT images, and interobserver agreement was calculated to evaluate SIJ fusion and anterior, posterior and entheseal bony bridging using 21 samples. The mean interobserver kappa coefficient agreement was 0.78 (95% confidence interval, 0.57–0.97), indicating substantial agreement. The whole spine CT data of ligaments, including anterior bony bridging of the vertebral body and spontaneous facet fusion, were also collected. Anterior bony bridging of the vertebral body was defined as candle-wax-type if it bulged > 3 mm from the anterior vertebral body wall and as smooth-type if it was < 3 mm (Fig. 1E). The number of anterior bony bridging of the vertebral body was evaluated in the cervical, thoracic (T) 1–T6, T7–T12, lumbar and whole spinal regions. Facet fusion was also classified into none, one-side and both sides (Fig. 1F).

### Statistical analysis

Differences between DISH and AS were analysed using the Mann–Whitney *U*-test for continuous variables and Fisher’s exact test for nominal variables. The JMP software version 12 (SAS Institute, Cary, North Carolina, USA) was used for all statistical analyses, and a *P*-value of < 0.05 was considered to indicate statistical significance.

## Results

### Characteristics of radiographic data

Table 1 shows the SIJ fusion, anterior, posterior and entheseal bony bridging analyses. Complete SIJ fusion was significantly higher in patients with AS than in those with DISH (AS: none 18.5%, one-side partial 0.0%, both sides partial 14.8%, one-side complete and another side partial 3.7% and both sides complete 63.0%; DISH: none 37.3%, one-side partial 21.8%, both sides partial 38.2%, one-side complete and another side partial 0.9% and both sides complete 1.8%; *P* < 0.001). Regarding SIJ, 63% of patients with DISH had a partial or complete fusion, whereas 19% of patients with AS did not demonstrate any evidence of SIJ fusion (Table 1). However, Anterior bony bridging (DISH: one-side 25.5% and both sides 41.8%; AS: one-side 0.0% and both sides 7.4%; *P* < 0.001) and posterior bridging (DISH: one-side 20.0% and both sides 16.4%; AS: one-side 11.1% and both sides 0.0%; *P* = 0.025) were higher in patients with DISH than those with AS (Table 1). After the age/sex matching, SIJ fusion was also significantly higher in patients with AS than those with DISH (*P* < 0.001), and anterior bony bridging (*P* < 0.001) was significantly higher in patients with DISH than those with AS (Table 2).

**Table 1:** Demographic data of patients.

Characteristic	DISH	AS	*P*
No. of patients	111	27	
Age, years ± SD	68.0 ± 11.8	47.0 ± 12.5	< 0.001*
Sex	Male 83, female 28	Male 21, Female 6	0.75
Radiographic data of sacroiliac joint			
Joint fusion %			< 0.001*
None	37.3	18.5	
One side partial	21.8	0.0	
Both sides partial	38.2	14.8	
One side complete and another side partial	0.9	3.7	
Both sides complete	1.8	63.0	
Anterior bony bridging %			< 0.001*
None	32.7	92.6	
One side	25.5	0.0	
Both sides	41.8	7.4	
Posterior bony bridging %			0.025*
None	63.6	88.9	
One side	20.0	11.1	
Both sides	16.4	0.0	
Entheseal bony bridging %			0.16
None	82.7	96.3	
One	6.4	3.7	
Both sides	10.9	0.0	

*DISH* Diffuse idiopathic skeletal hyperostosis, *AS* Ankylosing spondylitis, *SD* standard deviation.

*P < 0.05.

**Table 2:** Demographic data of patients after matching of age and sex.

Characteristic	DISH	AS	*P*
No. of patients	17	17	
Age, years ± SD	50.5 ± 10.3	50.5 ± 10.0	0.92
Sex	Male 13, female 4	Male 13, female 4	1.0
Radiographic data of sacroiliac joint after matching of age and sex			
Joint fusion %			< 0.001*
None	29.4	17.7	
One side partial	29.4	0.0	
Both sides partial	41.2	17.7	
One side complete and another side partial	0.0	5.9	
Both sides complete	0.0	58.8	
Anterior bony bridging %			< 0.001*
None	17.7	94.1	
One side	35.3	0.0	
Both sides	47.1	5.9	
Posterior bony bridging %			0.12
None	58.8	88.2	
One side	29.4	11.8	
Both sides	11.8	0.0	
Entheseal bony bridging %			0.60
None	88.2	94.1	
One	5.6	5.9	
Both sides	5.9	0.0	

*DISH* Diffuse idiopathic skeletal hyperostosis, *AS* Ankylosing spondylitis, *SD* standard deviation.

*P < 0.05.

Table 3 demonstrates the anterior bone bridging analysis on reconstructed spinal images. Anterior bony bridging was significantly higher in patients with AS than those with DISH in the lumbar (DISH: 0.4 ± 0.9, AS: 1.7 ± 2.2; *P* = 0.009). The percentage of candle-wax-type bone bridging was significantly higher in patients with DISH than those with AS (DISH: 0–30%, 11%; 30–70%, 10%; and 70–100%, 79%; AS: 0–30%, 79%; 30–70%, 21%; 70–100%, 0%; *P* < 0.001) (Table 3). This result was similar after age/sex matching (Table 3). Notably, 11% of patients with DISH had only 0–30% of candle-wax bridging, whereas up to 21% of patients with AS had 30–70% of candle-wax bridging.

**Table 3:** Radiographic data of anterior spinal bridging.

Characteristic	DISH	AS	*P*
Number of anterior bony bridging ± SD			
Cervical	1.2 ± 1.5	1.5 ± 2.5	0.35
Thoracic T1–6	3.8 ± 2.0	3.3 ± 2.5	0.59
Thoracic T7–12	3.7 ± 2.1	3.1 ± 2.6	0.57
Lumbar	0.4 ± 0.9	1.7 ± 2.2	0.009*
Whole spine	9.0 ± 4.4	9.6 ± 7.9	0.94
Percentage of candle wax type %			< 0.001*
0–30%	10.8	79.2	
30–70%	9.9	20.8	
70–100%	79.3	0.0	
Radiographic data of anterior spinal bridging after matching of age and sex			
Number of anterior bony bridging ± SD			
Cervical	1.2 ± 1.6	2.1 ± 2.8	0.71
Thoracic T1–6	4.0 ± 2.1	4.0 ± 2.1	0.87
Thoracic T7–12	3.0 ± 2.5	3.6 ± 2.7	0.33
Lumbar	0.4 ± 0.8	1.8 ± 2.2	0.082
Whole spine	8.6 ± 5.0	11.6 ± 8.1	0.29
Percentage of candle wax type %			< 0.001*
0–30%	17.7	70.6	
30–70%	5.9	29.4	
70–100%	76.5	0.0	

*DISH* Diffuse idiopathic skeletal hyperostosis, *AS* Ankylosing spondylitis, SD: standard deviation.

*P < 0.05.

Regarding the spinal facet fusion status, 83% of AS patients had facet fusion, whereas 61% of DISH patients had fusion. The number of both-side facet fusion was significantly higher in patients with AS than in those with DISH in T1–T6 (*P* = 0.004), either in T7–T12 (*P* < 0.001), in the lumbar spine (*P* < 0.001) and in the whole spine (*P* < 0.001) (Table 4). Conversely, the number of no facet fusion was higher in patients with DISH in T1–T6 (*P* = 0.002), T7–T12 (*P* < 0.001), lumbar (*P* < 0.001) and whole spine (*P* < 0.001) (Table 4). This result was similar after age/sex matching (Table 4).

**Table 4:** Radiographic data of spinal facet fusion.

Characteristic	DISH	AS	*P*
Cervical facet ± SD			
None	5.8 ± 2.2	5.1 ± 2.6	0.13
One side	0.1 ± 0.5	0.1 ± 0.4	0.83
Both sides	1.1 ± 2.2	1.6 ± 2.5	0.28
Thoracic T1–6 facet ± SD			
None	4.6 ± 1.9	2.9 ± 2.6	0.002*
One side	0.3 ± 0.5	0.1 ± 0.3	0.074
Both sides	1.1 ± 1.7	2.7 ± 2.6	0.004*
Thoracic T7–12 facet ± SD			
None	5.2 ± 1.7	2.7 ± 2.8	< 0.001*
One side	0.2 ± 0.5	0.1 ± 0.5	0.51
Both sides	0.6 ± 1.5	2.8 ± 2.8	< 0.001*
Lumbar facet ± SD			
None	4.8 ± 0.8	2.0 ± 2.3	< 0.001*
One side	0.0 ± 0.3	0.1 ± 0.4	0.78
Both sides	0.2 ± 0.7	2.4 ± 2.4	< 0.001*
Whole spine facet ± SD			
None	20.4 ± 4.6	13.0 ± 8.4	< 0.001*
One side	0.6 ± 1.0	0.4 ± 0.9	0.27
Both sides	3.1 ± 4.3	9.4 ± 8.5	< 0.001*
Radiographic data of spinal facet after matching of age and sex			
Cervical facet ± SD			
None	5.4 ± 2.7	4.4 ± 2.8	0.21
One side	0.0 ± 0.0	0.1 ± 0.2	0.35
Both sides	1.6 ± 2.7	2.4 ± 2.8	0.27
Thoracic T1–6 facet ± SD			
None	4.5 ± 1.7	2.4 ± 2.5	0.018*
One side	0.2 ± 0.4	0.1 ± 0.3	0.39
Both sides	1.3 ± 1.7	3.3 ± 2.5	0.028*
Thoracic T7–12 facet ± SD			
None	5.5 ± 1.0	2.5 ± 2.8	0.003*
One side	0.1 ± 0.2	0.2 ± 0.7	0.53
Both sides	0.4 ± 1.0	3.1 ± 2.8	0.004*
Lumbar facet ± SD			
None	4.9 ± 0.2	1.8 ± 2.2	< 0.001*
One side	0.0 ± 0.0	0.1 ± 0.5	0.35
Both sides	0.1 ± 0.2	2.6 ± 2.3	< 0.001*
Whole spine facet ± SD			
None	20.3 ± 3.7	11.4 ± 8.3	0.003*
One side	0.3 ± 0.5	0.5 ± 1.1	0.85
Both sides	3.4 ± 3.5	11.3 ± 8.5	0.009*

*DISH* Diffuse idiopathic skeletal hyperostosis, *AS* Ankylosing spondylitis, *SD* standard deviation.

*P < 0.05.

### Case presentation

**Figure 2:**
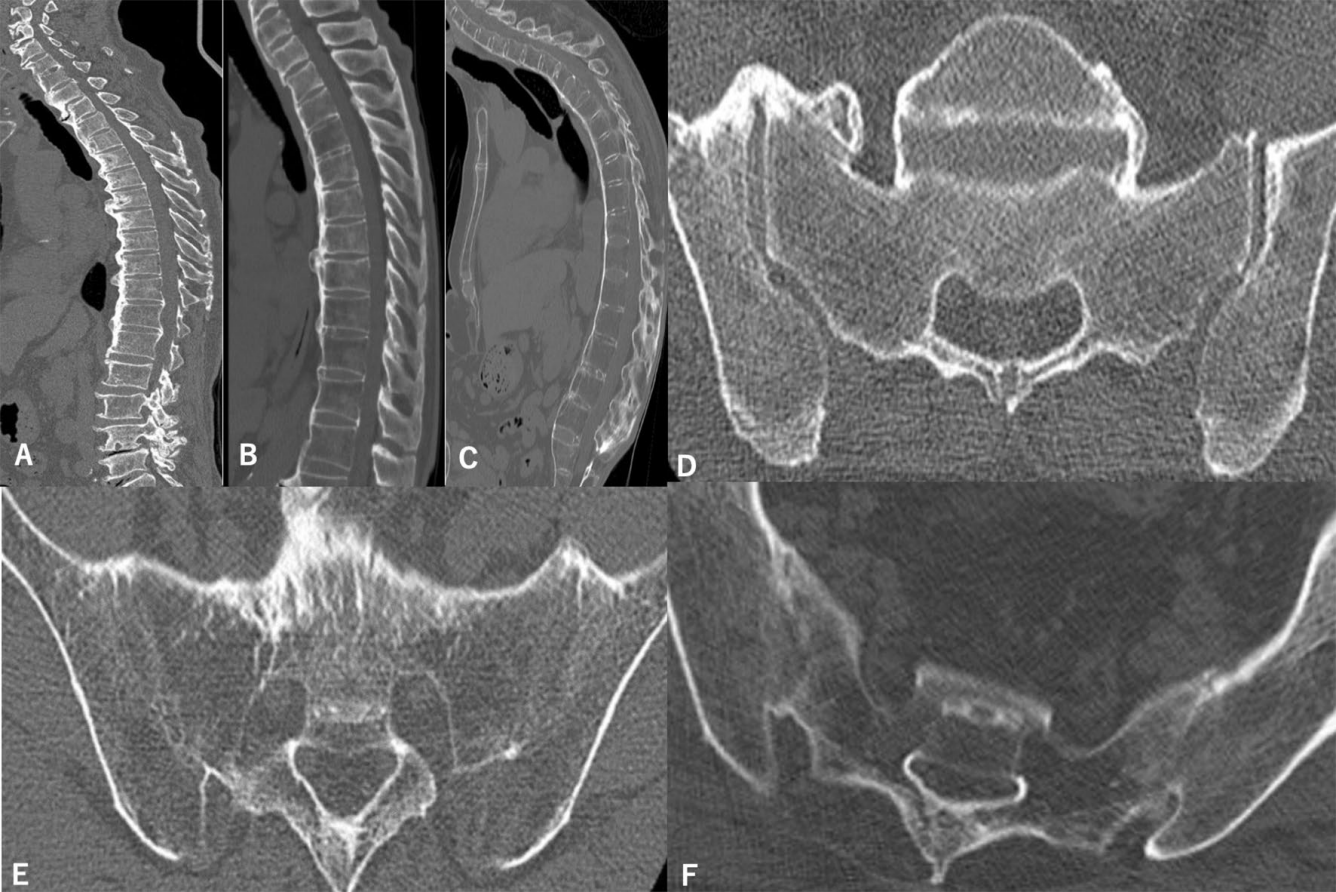
CT images of the case presentation. (**A**) Sagittal CT image of the whole spine shows 70–100% candle-wax-type in a 70-year-old male patient with DISH. (**B**) Sagittal CT image of the whole spine shows 0–30% candle-wax-type in an 82-year-old male patient with DISH. (**C**) Sagittal CT image of the whole spine shows 0–30% candle-wax-type in a 54-year-old male patient with AS. (**D**) Axial CT image of SIJ shows anterior bony bridging of the right side and no joint fusion in a 78-year-old male patient with DISH. (**E**) Axial CT image of the SIJ shows complete joint fusion in a 75-year-old male patient with DISH. (**F**) Axial CT image of the SIJ shows complete joint fusion in a 63-year-old male patient with AS.

A 70-year-old male patient showed a typical radiological feature of DISH: 70–100% candle-wax-type anterior bony bridging (Fig. 2A), whereas an 82-year-old male patient with DISH showed only 0–30% candle-wax-type bridging (Fig. 2B). A 54-year-old male patient with AS demonstrated typical features of AS 0–30% candle-wax-type (Fig. 2C). A 78-year-old male patient with DISH showed no SIJ fusion but anterior bony bridging of the right-sided SIJ (Fig. 2D), whereas a 75-year-old male patient with DISH had complete SIJ fusion (Fig. 2E). A 63-year-old male patient with AS showed typical complete SIJ fusion (Fig. 2F).

## Discussion

DISH is generally diagnosed using the Resnick criteria^7,8^, which are defined by swelling ossification of the anterior longitudinal ligament^1^ and absence of apophyseal joint ankylosis and SIJ erosion, sclerosis, or intraarticular osseous fusion^8^. Conversely, AS is often diagnosed using the modified New York criteria^14,21^. AS is characterised by annulus fibrosus ossification and adjacent vertebral body bridging anteriorly and laterally known as a ‘bamboo spine’^1^ and SIJ sclerosis, joint space narrowing, erosion, or fusion^1^. Some reports indicate that DISH and AS can be differentiated based on these radiological characteristics^1^. However, we sometimes encounter patients who do not have typical radiological findings defined in the abovementioned criteria and are difficult to differentiate between these two diseases. Therefore, this current study compared the detailed radiological characteristics of DISH and AS on spinopelvic CT images.

Previous literature has described that in the later phase of AS, SIJ shows sclerosis, joint space narrowing, erosion, or osseous fusion^1^. Conversely, in the SIJ of patients with DISH, only ligamentous area obliteration and mild synovial area narrowing can occur; however, SIJ fusion is not observed^1^. However, some other studies have reported that 23% of DISH is associated with fusion^15,19^, even though the DISH diagnosis does not require this involvement based on the Resnick criteria^8^. A previous study suggested that SIJ fusion occurring both in patients with DISH and AS may be possibly due to similar developmental pathways, leading to inflammation-associated enthesitis in younger patients in AS and more mechanistically associated enthesopathy in older patients with DISH^19^. In this study, as the percentage of partial and complete SIJ fusion was higher in AS than in DISH, up to 63% of patients with DISH had partial or complete SIJ fusion. The SIJ fusion rate in this study was larger than that in previous studies possibly due to the evaluation method in which we included partial SIJ fusion. However, this study demonstrated that SIJ fusion was frequently observed not only in patients with AS but in those with DISH and that might not be the necessary criteria to differentiate DISH from AS.

In the radiological evaluation of anterior and posterior bridging around the SIJ, previous studies have reported that patients with DISH have high anterior and posterior bridging rates around the SIJ. In their reports, 71.6%^15^, 48%^19^ and 30%^22^ of patients with DISH had anterior bony bridging and 5.4%^15^, 20%^19^ and 17%^22^ of patients with DISH had posterior bony bridging. In this study, we found that anterior and posterior bony bridging around the SIJ was significantly more common in DISH than in AS. We separately evaluated one-side and both-side bridging on the SIJ and found that both-side bridging was much higher in DISH than in AS either in anterior or posterior bridging. This finding may be useful for differentiating AS from DISH.

In the anterior spinal bony bridging of the vertebrae, inflammation occurs at the attachment of the annulus fibrosus, and the healing process results in AS syndesmophytes^1^. Conversely, bridging in DISH results from an ossification process involving the anterior longitudinal ligament^1^. Although both AS and DISH are characterised by anterior bony bridging, that of AS is generally characterised by smooth bridging ‘bamboo spine’ and that of DISH is by ossification of candle-wax-type ‘flowing mantles’^1^. We defined both of them as anterior bony bridging. The number of anterior spinal bony bridging was greater in AS than in DISH, especially in the lumbar spine. These results are similar after age and sex matching. As previously reported, DISH mainly affects the thoracic spine^18,23^. However, our study showed that AS tends to affect the lumbar spine, which may also be one of the differentiating points between DISH and AS.

In analysing the shape of spinal bridging, as expected, the rate of candle-wax-type was much higher in DISH than in AS. This result is consistent with that of a previous report^1^. However, a certain percentage of patients with DISH had surprisingly smooth-type bridging (0–30% candle-wax-type: 10.8%). Conversely, some patients with AS had candle-wax-type bridging (30–70% candle-wax-type: 20.8%). To the best of our knowledge, this is the first study to reveal that anterior bony bridging in patients with DISH occasionally demonstrated ‘AS-like’ bony bridging. Our findings refute that of the previously reported^1,5,8^, i.e. the typical appearance of bony bridging in DISH. Physicians should consider that candle-wax-type bridging is not always present in DISH, and this appearance alone cannot prove its diagnosis.

In evaluating the facet joint fusion, the DISH diagnosis requires the absence of joint fusion^8^. A previous study showed that facet joint ankylosis in AS is more common than that in DISH, and a small number of cervical ones with DISH were observed in whole-body magnetic resonance imaging^20^. They showed no thoracic and lumbar facet ankylosis in DISH. Another study reported that thoracic and lumbar facets in patients with AS had more inflammatory lesions^24^. We also found that both-side facet fusions of the thoracic, lumbar and whole spine were more common in AS than in DISH, and results were similar after age and sex matching (Table 4). Interestingly, 61% of patients with DISH had at least one facet fusion in our study. We first demonstrated that facet fusions occur at a high rate not only in AS but also in DISH. For the differential diagnosis of DISH and AS, spinal facet fusion exists should be considered in a certain number of patients with DISH in addition to SIJ fusion.

This study has some limitations. First, the sample size of patients with AS was relatively small because AS is uncommon in our country. However, most of the important comparisons reached statistically significant difference. Second, the number of bony bridging and fusion depends on the patient’s age. However, results after age matching showed a similar tendency. Third, although rare, there may be concurrent patients of AS and DISH, as reported in the past^10^. Fourth, we could not obtain functional from AS patients included in this study. Large-scale, prospective study is therefore needed to clarify relationships between clinical data and radiological findings.

In conclusion, both sides of complete SIJ fusion are common in patients with AS, and anterior/posterior bridging around the SIJ is common in patients with DISH. However, a considerable number of patients with DISH have SIJ fusion. In the anterior spinal bridging, patients with AS are characterised by smooth bridging, which commonly occurs in the lumbar spine of patients with AS when compared to those with DISH. Conversely, patients with DISH are characterised by candle-wax-type bridging, which commonly occurs in the thoracic spine. However, interestingly, a certain percentage of patients with DISH had smooth-type bridging and some patients with AS had candle-wax-type bridging. Furthermore, a considerable number of patients with DISH showed spinal facet fusion. These facts should be considered when making a diagnosis for AS or DISH.

## Data Availability

The datasets used and analyzed during the current study are available from the corresponding author on reasonable request.
